# Ethyl 1-[2-(1,3-benzoxazol-2-ylsulfan­yl)acet­yl]-4-hy­droxy-2,6-diphenyl-1,2,5,6-tetra­hydro­pyridine-3-carboxyl­ate

**DOI:** 10.1107/S1600536811022744

**Published:** 2011-06-18

**Authors:** G. Aridoss, S. Sundaramoorthy, D. Velmurugan, Y. T. Jeong

**Affiliations:** a Department of Image Science and Engineering, Pukyong National University, Busan 608-739, Republic of Korea; b Centre of Advanced Study in Crystallography and Biophysics, University of Madras, Guindy Campus, Chennai 600 025, India

## Abstract

In the title compound, C_29_H_26_N_2_O_5_S, the piperidine ring adopts a half-chair conformation. The phenyl rings are oriented at dihedral angles of 75.76 (12) and 86.64 (9)° with respect to the best plane through the piperidine ring. The dihedral angle between the two phenyl rings is 30.81 (13)°. The benzoxazole ring system is approximately planar [maximum deviation = 0.016 (4) Å]. The atoms of the ethyl side chain are disordered over two sets of sites [site occupancies = 0.376 (9) and 0.624 (9)]. The mol­ecular conformation is stabilized by an intra­molecular O—H⋯O hydrogen bond, generating an *S*(6) motif. The crystal packing is stabilized by inter­molecular C—H⋯O inter­actions, generating a chain running along the *a* axis.

## Related literature

For the synthesis and biological activity of piperidin-4-one-based amides, see: Aridoss *et al.* (2010*a* ▶). For related structures see: Aridoss *et al.* (2010*a* ▶,*b* ▶). For ring conformational analysis, see: Cremer & Pople (1975 ▶); Nardelli (1983 ▶).
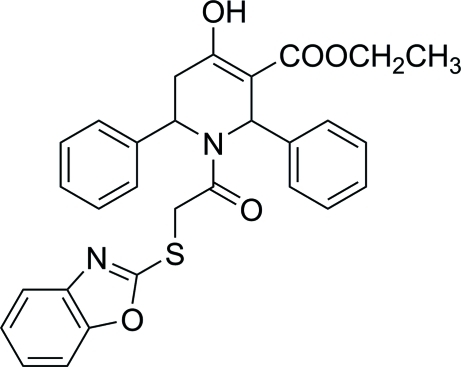


## Experimental

### 

#### Crystal data

C_29_H_26_N_2_O_5_S
*M* *_r_* = 514.58
Monoclinic, 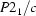

*a* = 12.4630 (2) Å
*b* = 24.3243 (5) Å
*c* = 9.2350 (2) Å
β = 109.608 (1)°
*V* = 2637.28 (9) Å^3^
*Z* = 4
Mo *K*α radiation
μ = 0.16 mm^−1^
*T* = 293 K
0.23 × 0.2 × 0.18 mm

#### Data collection

Bruker SMART APEXII area-detector diffractometer
Absorption correction: multi-scan (*SADABS*; Bruker, 2008 ▶) *T*_min_ = 0.963, *T*_max_ = 0.971
25589 measured reflections
6571 independent reflections
4020 reflections with *I* > 2σ(*I*)
*R*_int_ = 0.033

#### Refinement

*R*[*F* ^2^ > 2σ(*F* ^2^)] = 0.046
*wR*(*F* ^2^) = 0.128
*S* = 1.05
6571 reflections
343 parameters
1 restraint
H-atom parameters constrained
Δρ_max_ = 0.23 e Å^−3^
Δρ_min_ = −0.24 e Å^−3^

### 

Data collection: *APEX2* (Bruker, 2008 ▶); cell refinement: *SAINT* (Bruker, 2008 ▶); data reduction: *SAINT*; program(s) used to solve structure: *SHELXS97* (Sheldrick, 2008 ▶); program(s) used to refine structure: *SHELXL97* (Sheldrick, 2008 ▶); molecular graphics: *ORTEP-3* (Farrugia, 1997 ▶); software used to prepare material for publication: *SHELXL97* and *PLATON* (Spek, 2009 ▶).

## Supplementary Material

Crystal structure: contains datablock(s) global, I. DOI: 10.1107/S1600536811022744/lw2065sup1.cif

Structure factors: contains datablock(s) I. DOI: 10.1107/S1600536811022744/lw2065Isup2.hkl

Supplementary material file. DOI: 10.1107/S1600536811022744/lw2065Isup3.cml

Additional supplementary materials:  crystallographic information; 3D view; checkCIF report

## Figures and Tables

**Table 1:** Hydrogen-bond geometry (Å, °)

*D*—H⋯*A*	*D*—H	H⋯*A*	*D*⋯*A*	*D*—H⋯*A*
O1—H1*A*⋯O2	0.82	1.84	2.558 (2)	145
C13—H13⋯O2^i^	0.93	2.59	3.263 (2)	130

Symmetry code: (i) 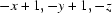.

**Fig. 1.**
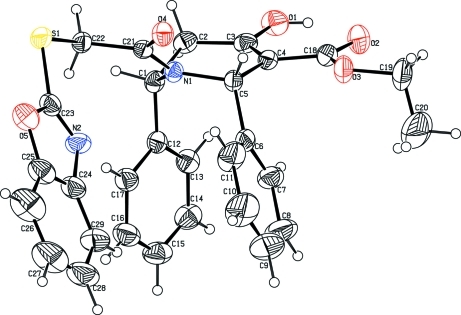
Perspective view of the molecule showing the thermal ellipsoids are drawn at 30% probability level.

**Fig. 2.**
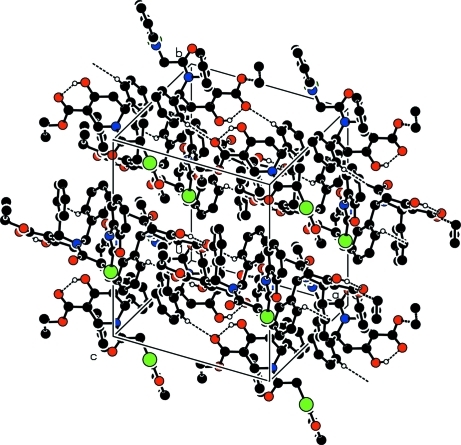
The crystal packing of the molecules viewed along c axis. For clarity, hydrogen atoms which are not involved in hydrogen bonding are omitted

## supplementary crystallographic information

### Comment

The *ORTEP* diagram of the title compound is shown in Fig. 1. The tetrahydropyridine ring adopts a half-chair conformation. The puckering parameters (Cremer & Pople, 1975) and the smallest displacement asymmetry parameters (Nardelli, 1983) for this ring are q2 = 0.3543 (18) Å, q3 = 0.2996 (18) Å, Q_T_ = 0.4639 (17)Å and θ = 49.8 (2)°, respectively. The phenyl rings are oriented at dihedral angles of 75.76 (12) and 86.64 (9)° with respect to the best plane through the piperidine ring. The dihedral angle between the two phenyl rings is 30.81 (13)°. The sum of the bond angles around the atom N1 [359.49 (4)°] of the tetrahydropyridine ring in the molecule is in accordance with *sp*^2^ hybridization.

The molecular structure is stabilized by a strong O—H···O hydrogen bond, wherein, atom O1 acts as a donor to O2, generating an *S*(6) motif. The crystal packing is stabilized by C—H···O intermolecular interaction which links the molecules into chain running along the *a* axis.

### Experimental

The title compound was prepared from *N*-bromoacetyl-3-carboxyethyl -2,6-diphenyl-4-hydroxy-Δ^3^-tetrahydropyridine and benzoxazol -2-thiol according to the literature method (Aridoss *et al.*, 2010*a*). Single crystals of the target molecule were obtained by the slow evaporation of its ethanolic solution at room temperature.

### Refinement

The C atoms of the ethyl side chain are disordered over two positions (C19/C19' and C20/C20') with refined occupancies of 0.376 (9) and 0.624 (9). The corresponding bond distances involving the disordered atoms were restrained to be equal and also the same U^ij^ parameters were used for atoms C19/C19' and C20/C20'. All H atoms were fixed geometrically and allowed to ride on their parent C atoms, with C—H distances fixed in the range 0.93–0.97 Å with *U*_iso_(H) = 1.5*U*_eq_(C) for methyl H and 1.2*U*_eq_(C) for other H atoms.

### Crystal data

**Table:**

C_29_H_26_N_2_O_5_S	*F*(000) = 1080
*M**_r_* = 514.58	*D*_x_ = 1.296 Mg m^−^^3^
Monoclinic, *P*2_1_/*c*	Mo *K*α radiation, λ = 0.71073 Å
Hall symbol: -P 2ybc	Cell parameters from 1535 reflections
*a* = 12.4630 (2) Å	θ = 1.7–28.3°
*b* = 24.3243 (5) Å	µ = 0.16 mm^−^^1^
*c* = 9.2350 (2) Å	*T* = 293 K
β = 109.608 (1)°	Block, colourless
*V* = 2637.28 (9) Å^3^	0.23 × 0.2 × 0.18 mm
*Z* = 4	

### Data collection

**Table:**

Bruker SMART APEXII area-detector diffractometer	6571 independent reflections
Radiation source: fine-focus sealed tube	4020 reflections with *I* > 2σ(*I*)
graphite	*R*_int_ = 0.033
ω and φ scans	θ_max_ = 28.3°, θ_min_ = 1.7°
Absorption correction: multi-scan (*SADABS*; Bruker, 2008)	*h* = −16→14
*T*_min_ = 0.963, *T*_max_ = 0.971	*k* = −32→30
25589 measured reflections	*l* = −11→12

### Refinement

**Table:**

Refinement on *F*^2^	Primary atom site location: structure-invariant direct methods
Least-squares matrix: full	Secondary atom site location: difference Fourier map
*R*[*F*^2^ > 2σ(*F*^2^)] = 0.046	Hydrogen site location: inferred from neighbouring sites
*wR*(*F*^2^) = 0.128	H-atom parameters constrained
*S* = 1.05	*w* = 1/[σ^2^(*F*_o_^2^) + (0.0552*P*)^2^ + 0.311*P*] where *P* = (*F*_o_^2^ + 2*F*_c_^2^)/3
6571 reflections	(Δ/σ)_max_ = 0.001
343 parameters	Δρ_max_ = 0.23 e Å^−^^3^
1 restraint	Δρ_min_ = −0.24 e Å^−^^3^

### Special details

**Table:**

Geometry. All e.s.d.'s (except the e.s.d. in the dihedral angle between two l.s. planes) are estimated using the full covariance matrix. The cell e.s.d.'s are taken into account individually in the estimation of e.s.d.'s in distances, angles and torsion angles; correlations between e.s.d.'s in cell parameters are only used when they are defined by crystal symmetry. An approximate (isotropic) treatment of cell e.s.d.'s is used for estimating e.s.d.'s involving l.s. planes.
Refinement. Refinement of *F*^2^ against ALL reflections. The weighted *R*-factor *wR* and goodness of fit *S* are based on *F*^2^, conventional *R*-factors *R* are based on *F*, with *F* set to zero for negative *F*^2^. The threshold expression of *F*^2^ > σ(*F*^2^) is used only for calculating *R*-factors(gt) *etc*. and is not relevant to the choice of reflections for refinement. *R*-factors based on *F*^2^ are statistically about twice as large as those based on *F*, and *R*- factors based on ALL data will be even larger.

### Fractional atomic coordinates and isotropic or equivalent isotropic displacement parameters (Å^2^)

**Table:**

	*x*	*y*	*z*	*U*_iso_*/*U*_eq_	Occ. (<1)
C1	0.88932 (13)	0.51361 (7)	0.12658 (18)	0.0442 (4)	
H1	0.9663	0.5002	0.1408	0.053*	
C2	0.81770 (15)	0.46363 (7)	0.1329 (2)	0.0512 (4)	
H2A	0.7993	0.4435	0.0369	0.061*	
H2B	0.8616	0.4395	0.2150	0.061*	
C3	0.71060 (14)	0.47926 (7)	0.15925 (19)	0.0490 (4)	
C4	0.69728 (13)	0.52718 (7)	0.22370 (19)	0.0450 (4)	
C5	0.78980 (13)	0.57022 (6)	0.27182 (19)	0.0422 (4)	
H5	0.8040	0.5773	0.3811	0.051*	
C6	0.75751 (15)	0.62540 (7)	0.1898 (2)	0.0519 (4)	
C7	0.67108 (18)	0.63139 (9)	0.0521 (2)	0.0665 (5)	
H7	0.6299	0.6007	0.0042	0.080*	
C8	0.6440 (3)	0.68249 (12)	−0.0170 (3)	0.1006 (9)	
H8	0.5857	0.6860	−0.1109	0.121*	
C9	0.7038 (3)	0.72767 (13)	0.0541 (5)	0.1234 (12)	
H9	0.6859	0.7621	0.0085	0.148*	
C10	0.7896 (3)	0.72256 (11)	0.1916 (5)	0.1218 (11)	
H10	0.8301	0.7535	0.2393	0.146*	
C11	0.8165 (2)	0.67160 (9)	0.2602 (3)	0.0862 (7)	
H11	0.8746	0.6684	0.3544	0.103*	
C12	0.85080 (14)	0.54573 (7)	−0.02325 (19)	0.0457 (4)	
C13	0.75318 (15)	0.53281 (8)	−0.1447 (2)	0.0553 (5)	
H13	0.7080	0.5035	−0.1352	0.066*	
C14	0.72199 (19)	0.56263 (9)	−0.2793 (2)	0.0685 (6)	
H14	0.6555	0.5538	−0.3589	0.082*	
C15	0.7888 (2)	0.60518 (9)	−0.2957 (2)	0.0739 (6)	
H15	0.7682	0.6251	−0.3870	0.089*	
C16	0.88687 (19)	0.61857 (9)	−0.1767 (3)	0.0710 (6)	
H16	0.9327	0.6473	−0.1880	0.085*	
C17	0.91674 (16)	0.58939 (8)	−0.0412 (2)	0.0567 (5)	
H17	0.9820	0.5991	0.0393	0.068*	
C18	0.59236 (14)	0.53668 (9)	0.2550 (2)	0.0567 (5)	
C19	0.4825 (7)	0.6007 (8)	0.335 (2)	0.139 (5)	0.376 (9)
H19A	0.4214	0.5893	0.2437	0.167*	0.376 (9)
H19B	0.4691	0.5857	0.4250	0.167*	0.376 (9)
C19'	0.4874 (4)	0.6022 (5)	0.3484 (7)	0.0873 (18)	0.624 (9)
H19C	0.4396	0.5707	0.3491	0.105*	0.624 (9)
H19D	0.5043	0.6211	0.4460	0.105*	0.624 (9)
C20	0.4919 (7)	0.6599 (4)	0.3442 (17)	0.139 (5)	0.376 (9)
H20A	0.5441	0.6704	0.4430	0.209*	0.376 (9)
H20B	0.4184	0.6756	0.3303	0.209*	0.376 (9)
H20C	0.5194	0.6731	0.2651	0.209*	0.376 (9)
C20'	0.4273 (4)	0.6389 (2)	0.2241 (5)	0.0873 (18)	0.624 (9)
H20D	0.4759	0.6692	0.2210	0.131*	0.624 (9)
H20E	0.3601	0.6525	0.2406	0.131*	0.624 (9)
H20F	0.4062	0.6193	0.1283	0.131*	0.624 (9)
C21	0.99471 (13)	0.55658 (6)	0.38113 (19)	0.0401 (4)	
C22	1.10534 (13)	0.53591 (7)	0.3643 (2)	0.0474 (4)	
H22A	1.1021	0.5412	0.2588	0.057*	
H22B	1.1121	0.4968	0.3855	0.057*	
C23	1.20310 (15)	0.63370 (7)	0.4034 (2)	0.0514 (4)	
C24	1.13948 (18)	0.70527 (8)	0.2721 (3)	0.0639 (5)	
C25	1.2373 (2)	0.71956 (8)	0.3851 (3)	0.0685 (6)	
C26	1.2833 (3)	0.77171 (11)	0.4042 (4)	0.1052 (9)	
H26	1.3514	0.7803	0.4813	0.126*	
C27	1.2207 (4)	0.80994 (11)	0.3007 (5)	0.1154 (11)	
H27	1.2474	0.8459	0.3081	0.138*	
C28	1.1209 (3)	0.79739 (11)	0.1875 (4)	0.1077 (10)	
H28	1.0810	0.8249	0.1213	0.129*	
C29	1.0783 (2)	0.74448 (10)	0.1695 (3)	0.0895 (7)	
H29	1.0109	0.7356	0.0914	0.107*	
N1	0.89780 (10)	0.54863 (5)	0.26052 (15)	0.0399 (3)	
N2	1.11968 (13)	0.64910 (6)	0.2867 (2)	0.0625 (4)	
O1	0.63128 (11)	0.43949 (5)	0.11907 (15)	0.0671 (4)	
H1A	0.5752	0.4492	0.1399	0.101*	
O2	0.51125 (11)	0.50477 (6)	0.21915 (18)	0.0791 (4)	
O3	0.59263 (10)	0.58358 (6)	0.32943 (16)	0.0657 (4)	
O4	0.99521 (9)	0.57867 (5)	0.50028 (14)	0.0515 (3)	
O5	1.28120 (11)	0.67320 (6)	0.47306 (16)	0.0682 (4)	
S1	1.22926 (4)	0.56980 (2)	0.48965 (6)	0.05622 (15)	

### Atomic displacement parameters (Å^2^)

**Table:**

	*U*^11^	*U*^22^	*U*^33^	*U*^12^	*U*^13^	*U*^23^
C1	0.0438 (9)	0.0380 (9)	0.0522 (9)	0.0028 (7)	0.0179 (7)	−0.0050 (7)
C2	0.0582 (10)	0.0370 (9)	0.0556 (10)	0.0000 (8)	0.0153 (8)	−0.0023 (8)
C3	0.0471 (9)	0.0430 (10)	0.0487 (9)	−0.0077 (8)	0.0051 (8)	0.0074 (8)
C4	0.0364 (8)	0.0479 (10)	0.0479 (9)	−0.0003 (7)	0.0105 (7)	0.0088 (8)
C5	0.0376 (8)	0.0429 (9)	0.0481 (9)	0.0010 (7)	0.0171 (7)	−0.0014 (7)
C6	0.0500 (10)	0.0413 (10)	0.0728 (12)	0.0085 (8)	0.0316 (9)	0.0024 (9)
C7	0.0801 (14)	0.0556 (13)	0.0687 (13)	0.0182 (10)	0.0313 (11)	0.0113 (10)
C8	0.128 (2)	0.0773 (19)	0.1017 (19)	0.0435 (18)	0.0454 (17)	0.0357 (16)
C9	0.147 (3)	0.0582 (19)	0.180 (4)	0.0325 (19)	0.075 (3)	0.044 (2)
C10	0.116 (2)	0.0432 (15)	0.205 (4)	−0.0003 (15)	0.052 (2)	0.0068 (19)
C11	0.0755 (15)	0.0451 (13)	0.133 (2)	0.0010 (11)	0.0281 (14)	−0.0042 (13)
C12	0.0493 (9)	0.0432 (10)	0.0493 (10)	0.0051 (8)	0.0228 (8)	−0.0043 (8)
C13	0.0589 (11)	0.0577 (12)	0.0508 (10)	0.0008 (9)	0.0202 (9)	−0.0053 (9)
C14	0.0766 (14)	0.0754 (15)	0.0498 (11)	0.0106 (12)	0.0163 (10)	−0.0018 (10)
C15	0.1025 (17)	0.0696 (15)	0.0538 (12)	0.0213 (13)	0.0318 (12)	0.0106 (11)
C16	0.0895 (15)	0.0556 (13)	0.0783 (15)	0.0004 (11)	0.0418 (13)	0.0099 (11)
C17	0.0603 (11)	0.0507 (11)	0.0621 (11)	−0.0005 (9)	0.0244 (9)	0.0013 (9)
C18	0.0408 (9)	0.0696 (13)	0.0564 (11)	0.0021 (9)	0.0121 (8)	0.0154 (10)
C19	0.060 (4)	0.129 (7)	0.253 (11)	−0.009 (4)	0.084 (6)	−0.055 (7)
C19'	0.067 (2)	0.126 (4)	0.074 (2)	0.0404 (19)	0.0303 (17)	0.0150 (18)
C20	0.060 (4)	0.129 (7)	0.253 (11)	−0.009 (4)	0.084 (6)	−0.055 (7)
C20'	0.067 (2)	0.126 (4)	0.074 (2)	0.0404 (19)	0.0303 (17)	0.0150 (18)
C21	0.0398 (8)	0.0328 (8)	0.0483 (9)	−0.0017 (6)	0.0156 (7)	0.0060 (7)
C22	0.0414 (9)	0.0384 (9)	0.0632 (11)	0.0018 (7)	0.0187 (8)	0.0064 (8)
C23	0.0487 (10)	0.0480 (10)	0.0617 (11)	−0.0084 (8)	0.0240 (9)	0.0026 (9)
C24	0.0726 (13)	0.0475 (12)	0.0816 (14)	−0.0018 (10)	0.0390 (11)	0.0110 (10)
C25	0.0927 (16)	0.0431 (11)	0.0836 (15)	−0.0089 (11)	0.0482 (13)	−0.0039 (11)
C26	0.142 (2)	0.0591 (17)	0.128 (2)	−0.0321 (17)	0.062 (2)	−0.0240 (16)
C27	0.174 (3)	0.0439 (16)	0.161 (3)	−0.009 (2)	0.101 (3)	−0.0040 (19)
C28	0.153 (3)	0.0565 (17)	0.150 (3)	0.0250 (18)	0.099 (2)	0.0374 (18)
C29	0.1020 (18)	0.0645 (15)	0.1127 (19)	0.0125 (13)	0.0501 (15)	0.0317 (14)
N1	0.0372 (7)	0.0358 (7)	0.0476 (8)	0.0018 (6)	0.0154 (6)	−0.0017 (6)
N2	0.0595 (9)	0.0480 (10)	0.0755 (11)	−0.0059 (8)	0.0168 (8)	0.0175 (8)
O1	0.0599 (8)	0.0576 (8)	0.0738 (9)	−0.0203 (6)	0.0092 (7)	0.0000 (7)
O2	0.0411 (7)	0.0926 (11)	0.0999 (11)	−0.0139 (7)	0.0189 (7)	0.0082 (9)
O3	0.0448 (7)	0.0847 (10)	0.0738 (9)	0.0084 (7)	0.0280 (6)	0.0025 (8)
O4	0.0459 (6)	0.0572 (8)	0.0505 (7)	−0.0026 (5)	0.0148 (5)	−0.0073 (6)
O5	0.0700 (9)	0.0599 (9)	0.0748 (9)	−0.0195 (7)	0.0246 (7)	−0.0099 (7)
S1	0.0396 (2)	0.0558 (3)	0.0685 (3)	−0.0013 (2)	0.0118 (2)	0.0148 (2)

### Geometric parameters (Å, °)

**Table:**

C1—N1	1.476 (2)	C18—O3	1.331 (2)
C1—C12	1.520 (2)	C19—C20	1.44 (2)
C1—C2	1.521 (2)	C19—O3	1.453 (3)
C1—H1	0.9800	C19—H19A	0.9700
C2—C3	1.484 (2)	C19—H19B	0.9700
C2—H2A	0.9700	C19'—C20'	1.449 (9)
C2—H2B	0.9700	C19'—O3	1.453 (2)
C3—O1	1.3434 (19)	C19'—H19C	0.9700
C3—C4	1.344 (2)	C19'—H19D	0.9700
C4—C18	1.449 (2)	C20—H20A	0.9600
C4—C5	1.510 (2)	C20—H20B	0.9600
C5—N1	1.4807 (19)	C20—H20C	0.9600
C5—C6	1.527 (2)	C20'—H20D	0.9600
C5—H5	0.9800	C20'—H20E	0.9600
C6—C7	1.371 (3)	C20'—H20F	0.9600
C6—C11	1.380 (3)	C21—O4	1.2226 (19)
C7—C8	1.386 (3)	C21—N1	1.354 (2)
C7—H7	0.9300	C21—C22	1.524 (2)
C8—C9	1.366 (4)	C22—S1	1.7901 (17)
C8—H8	0.9300	C22—H22A	0.9700
C9—C10	1.364 (4)	C22—H22B	0.9700
C9—H9	0.9300	C23—N2	1.277 (2)
C10—C11	1.381 (4)	C23—O5	1.365 (2)
C10—H10	0.9300	C23—S1	1.7271 (19)
C11—H11	0.9300	C24—C25	1.357 (3)
C12—C13	1.386 (2)	C24—C29	1.380 (3)
C12—C17	1.387 (2)	C24—N2	1.403 (2)
C13—C14	1.377 (3)	C25—C26	1.379 (3)
C13—H13	0.9300	C25—O5	1.390 (3)
C14—C15	1.369 (3)	C26—C27	1.374 (4)
C14—H14	0.9300	C26—H26	0.9300
C15—C16	1.381 (3)	C27—C28	1.363 (4)
C15—H15	0.9300	C27—H27	0.9300
C16—C17	1.376 (3)	C28—C29	1.381 (4)
C16—H16	0.9300	C28—H28	0.9300
C17—H17	0.9300	C29—H29	0.9300
C18—O2	1.229 (2)	O1—H1A	0.8200
			
N1—C1—C12	112.22 (13)	O2—C18—C4	124.2 (2)
N1—C1—C2	107.88 (13)	O3—C18—C4	113.17 (16)
C12—C1—C2	115.83 (14)	C20—C19—O3	103.2 (10)
N1—C1—H1	106.8	C20—C19—H19A	111.1
C12—C1—H1	106.8	O3—C19—H19A	111.1
C2—C1—H1	106.8	C20—C19—H19B	111.1
C3—C2—C1	111.90 (14)	O3—C19—H19B	111.1
C3—C2—H2A	109.2	H19A—C19—H19B	109.1
C1—C2—H2A	109.2	C20'—C19'—O3	110.1 (4)
C3—C2—H2B	109.2	C20'—C19'—H19C	109.6
C1—C2—H2B	109.2	O3—C19'—H19C	109.6
H2A—C2—H2B	107.9	C20'—C19'—H19D	109.6
O1—C3—C4	123.92 (16)	O3—C19'—H19D	109.6
O1—C3—C2	112.90 (15)	H19C—C19'—H19D	108.2
C4—C3—C2	123.11 (15)	C19'—C20'—H20D	109.5
C3—C4—C18	118.71 (16)	C19'—C20'—H20E	109.5
C3—C4—C5	122.42 (14)	H20D—C20'—H20E	109.5
C18—C4—C5	118.80 (16)	C19'—C20'—H20F	109.5
N1—C5—C4	110.84 (13)	H20D—C20'—H20F	109.5
N1—C5—C6	111.79 (13)	H20E—C20'—H20F	109.5
C4—C5—C6	114.55 (13)	O4—C21—N1	122.46 (14)
N1—C5—H5	106.4	O4—C21—C22	120.34 (14)
C4—C5—H5	106.4	N1—C21—C22	117.19 (14)
C6—C5—H5	106.4	C21—C22—S1	113.33 (12)
C7—C6—C11	118.61 (19)	C21—C22—H22A	108.9
C7—C6—C5	123.21 (17)	S1—C22—H22A	108.9
C11—C6—C5	118.16 (18)	C21—C22—H22B	108.9
C6—C7—C8	121.1 (2)	S1—C22—H22B	108.9
C6—C7—H7	119.5	H22A—C22—H22B	107.7
C8—C7—H7	119.5	N2—C23—O5	116.17 (16)
C9—C8—C7	119.4 (3)	N2—C23—S1	128.87 (14)
C9—C8—H8	120.3	O5—C23—S1	114.93 (13)
C7—C8—H8	120.3	C25—C24—C29	119.9 (2)
C10—C9—C8	120.3 (3)	C25—C24—N2	108.47 (18)
C10—C9—H9	119.8	C29—C24—N2	131.7 (2)
C8—C9—H9	119.8	C24—C25—C26	124.2 (2)
C9—C10—C11	120.2 (3)	C24—C25—O5	108.52 (17)
C9—C10—H10	119.9	C26—C25—O5	127.3 (2)
C11—C10—H10	119.9	C27—C26—C25	114.7 (3)
C6—C11—C10	120.4 (3)	C27—C26—H26	122.6
C6—C11—H11	119.8	C25—C26—H26	122.6
C10—C11—H11	119.8	C28—C27—C26	122.8 (3)
C13—C12—C17	118.11 (17)	C28—C27—H27	118.6
C13—C12—C1	122.93 (16)	C26—C27—H27	118.6
C17—C12—C1	118.95 (15)	C27—C28—C29	121.1 (3)
C14—C13—C12	121.07 (19)	C27—C28—H28	119.5
C14—C13—H13	119.5	C29—C28—H28	119.5
C12—C13—H13	119.5	C24—C29—C28	117.4 (3)
C15—C14—C13	120.0 (2)	C24—C29—H29	121.3
C15—C14—H14	120.0	C28—C29—H29	121.3
C13—C14—H14	120.0	C21—N1—C1	124.39 (13)
C14—C15—C16	119.9 (2)	C21—N1—C5	118.19 (13)
C14—C15—H15	120.0	C1—N1—C5	116.91 (12)
C16—C15—H15	120.0	C23—N2—C24	104.33 (17)
C17—C16—C15	119.9 (2)	C3—O1—H1A	109.5
C17—C16—H16	120.0	C18—O3—C19'	118.9 (5)
C15—C16—H16	120.0	C18—O3—C19	115.3 (7)
C16—C17—C12	120.89 (19)	C19'—O3—C19	4.8 (9)
C16—C17—H17	119.6	C23—O5—C25	102.52 (15)
C12—C17—H17	119.6	C23—S1—C22	97.35 (8)
O2—C18—O3	122.67 (17)		
			
N1—C1—C2—C3	48.57 (18)	C29—C24—C25—C26	−1.5 (3)
C12—C1—C2—C3	−78.14 (18)	N2—C24—C25—C26	179.3 (2)
C1—C2—C3—O1	160.73 (14)	C29—C24—C25—O5	179.21 (19)
C1—C2—C3—C4	−22.4 (2)	N2—C24—C25—O5	0.0 (2)
O1—C3—C4—C18	1.2 (2)	C24—C25—C26—C27	1.5 (4)
C2—C3—C4—C18	−175.36 (15)	O5—C25—C26—C27	−179.4 (2)
O1—C3—C4—C5	178.08 (15)	C25—C26—C27—C28	−0.1 (4)
C2—C3—C4—C5	1.5 (3)	C26—C27—C28—C29	−1.1 (5)
C3—C4—C5—N1	−8.7 (2)	C25—C24—C29—C28	0.2 (3)
C18—C4—C5—N1	168.15 (13)	N2—C24—C29—C28	179.2 (2)
C3—C4—C5—C6	118.88 (17)	C27—C28—C29—C24	1.1 (4)
C18—C4—C5—C6	−64.23 (19)	O4—C21—N1—C1	−168.31 (15)
N1—C5—C6—C7	106.37 (18)	C22—C21—N1—C1	10.7 (2)
C4—C5—C6—C7	−20.8 (2)	O4—C21—N1—C5	3.2 (2)
N1—C5—C6—C11	−75.4 (2)	C22—C21—N1—C5	−177.75 (13)
C4—C5—C6—C11	157.43 (17)	C12—C1—N1—C21	−120.16 (16)
C11—C6—C7—C8	1.0 (3)	C2—C1—N1—C21	111.06 (16)
C5—C6—C7—C8	179.19 (19)	C12—C1—N1—C5	68.23 (17)
C6—C7—C8—C9	−0.6 (4)	C2—C1—N1—C5	−60.56 (17)
C7—C8—C9—C10	0.2 (5)	C4—C5—N1—C21	−132.40 (14)
C8—C9—C10—C11	−0.2 (5)	C6—C5—N1—C21	98.49 (17)
C7—C6—C11—C10	−0.9 (3)	C4—C5—N1—C1	39.75 (18)
C5—C6—C11—C10	−179.2 (2)	C6—C5—N1—C1	−89.36 (17)
C9—C10—C11—C6	0.5 (5)	O5—C23—N2—C24	−0.5 (2)
N1—C1—C12—C13	−120.99 (16)	S1—C23—N2—C24	177.45 (15)
C2—C1—C12—C13	3.5 (2)	C25—C24—N2—C23	0.3 (2)
N1—C1—C12—C17	60.14 (19)	C29—C24—N2—C23	−178.8 (2)
C2—C1—C12—C17	−175.36 (15)	O2—C18—O3—C19'	−9.8 (4)
C17—C12—C13—C14	−0.4 (3)	C4—C18—O3—C19'	171.1 (3)
C1—C12—C13—C14	−179.27 (16)	O2—C18—O3—C19	−13.3 (10)
C12—C13—C14—C15	1.2 (3)	C4—C18—O3—C19	167.7 (10)
C13—C14—C15—C16	−0.7 (3)	C20'—C19'—O3—C18	−93.0 (8)
C14—C15—C16—C17	−0.5 (3)	C20'—C19'—O3—C19	−52 (15)
C15—C16—C17—C12	1.3 (3)	C20—C19—O3—C18	−151.2 (9)
C13—C12—C17—C16	−0.9 (3)	C20—C19—O3—C19'	69 (15)
C1—C12—C17—C16	178.06 (16)	N2—C23—O5—C25	0.5 (2)
C3—C4—C18—O2	−3.9 (3)	S1—C23—O5—C25	−177.77 (13)
C5—C4—C18—O2	179.06 (17)	C24—C25—O5—C23	−0.2 (2)
C3—C4—C18—O3	175.12 (15)	C26—C25—O5—C23	−179.5 (2)
C5—C4—C18—O3	−1.9 (2)	N2—C23—S1—C22	−0.05 (19)
O4—C21—C22—S1	−23.5 (2)	O5—C23—S1—C22	177.90 (13)
N1—C21—C22—S1	157.47 (12)	C21—C22—S1—C23	−68.22 (13)

### Hydrogen-bond geometry (Å, °)

**Table:**

*D*—H···*A*	*D*—H	H···*A*	*D*···*A*	*D*—H···*A*
O1—H1A···O2	0.82	1.84	2.558 (2)	145
C13—H13···O2^i^	0.93	2.59	3.263 (2)	130

Symmetry codes: (i) −*x*+1, −*y*+1, −*z*.

## References

[bb1] Aridoss, G., Amirthaganesan, S. & Jeong, Y. T. (2010*a*). *Bioorg. Med. Chem. Lett.* **20**, 2242–2249. 10.1016/j.bmcl.2010.02.015 20207140

[bb2] Aridoss, G., Sundaramoorthy, S., Velmurugan, D., Park, K. S. & Jeong, Y. T. (2010*b*). *Acta Cryst.* E**66**, o1982. 10.1107/S1600536810026413 PMC3007352 21588298

[bb3] Bruker (2008). *APEX2*, *SAINT* and *SADABS* Bruker AXS Inc., Madison, Wisconsin, USA.

[bb4] Cremer, D. & Pople, J. A. (1975). *J. Am. Chem. Soc.* **97**, 1354–1358.

[bb5] Farrugia, L. J. (1997). *J. Appl. Cryst.* **30**, 565.

[bb6] Nardelli, M. (1983). *Acta Cryst.* C**39**, 1141–1142.

[bb7] Sheldrick, G. M. (2008). *Acta Cryst.* A**64**, 112–122. 10.1107/S0108767307043930 18156677

[bb8] Spek, A. L. (2009). *Acta Cryst.* D**65**, 148–155. 10.1107/S090744490804362X PMC2631630 19171970

